# Comparison of classical and patient-preferred music on anxiety and recovery after ınguinal hernia repair: a prospective randomized controlled study

**DOI:** 10.1186/s13741-024-00434-3

**Published:** 2024-08-14

**Authors:** Fatma Kavak Akelma, Savaş Altınsoy, Burak Nalbant, Derya Özkan, Jülide Ergil

**Affiliations:** 1 Department of Anesthesiology and Reanimation, Faculty of Medicine, Ankara Yıldırım Beyazıt University, Ankara, Turkey; 2 Department of Anesthesiology and Reanimation, Ankara Bilkent City Hospital, Ankara, Turkey; 3 Department of Anesthesiology and Reanimation, Faculty of Medicine, University of Health Sciences,, Ankara, Turkey; 4 Department of Anesthesiology and Reanimation, Ankara Etlik City Hospital, Ankara, Turkey

**Keywords:** Music, Favorite, Postoperative pain, Patient satisfaction, QoR-40, Hemodynamics

## Abstract

**Background:**

We aimed to evaluate the effects of preoperative listening to patient-preferred music and classical music on postoperative anxiety and recovery.

**Methods:**

A prospective, randomized controlled, single-blind study included 255 patients who were scheduled for elective inguinal hernia operation under general anesthesia. Spielberger state State-Trait Anxiety Inventory form 1,2 (STAI-I, STAI-II), Quality of Recovery Score-40 (QoR-40) were applied in the preoperatively. In the preoperative period, the preferential music group (group P) patients listened to their favorite music, while patients in the classical music group (group C) listened to classical music, music was not played in the control group (group N). STAI-I, QoR-40 questionnaire, pain status, and patient satisfaction in the postoperative period were recorded by a blinded investigator.

**Results:**

A total of 217 patients participated in the study analysis. Postoperative STAI-1 score was lower in group P than in group N (*p* = 0.025) and was similar among other groups. The postoperative QoR-40 score was significantly higher in group P than in group N (*p* = 0.003), and it was similar between the other groups. While SBP, DBP and HR premusic and post-music changes were significant, there was no difference in other groups. There was no difference between the groups in the NRS score. The patient satisfaction score was significantly higher in group P.

**Conclusions:**

Preoperative patient-preferred music application reduces postoperative anxiety and improves recovery quality compared to classical music. In addition, regulation of hemodynamic data and patient satisfaction increase in a preferential music application, but pain scores do not change.

**Trial registration:**

NCT04277559|https://www.clinicaltrials.gov/

## Introduction

People often experience high levels of anxiety while awaiting surgical procedures (Bradt et al. 2013). This preoperative anxiety is defined as an unpleasant state of discomfort or nervousness resulting from the patient’s concern about hospitalization, anesthesia, surgery, or an unknown procedure (Ramsay 1972). Preoperative anxiety can lead to activation of the sympathetic nervous system, resulting in adverse hemodynamic responses such as increased blood pressure and heart rate, increased postoperative pain, increased incidence of sleep disturbance, delayed wound healing, increased risk of infection, and delayed postoperative recovery and discharge (Bradt et al. 2013; Kaur et al. 2022; Jawaid et al. 2007; McClurkin and Smith 2016). Preoperative and associated postoperative anxiety is recognized as a preventable risk factor that can reduce postoperative complications (Britteon et al. 2022; Stamenkovic et al. 2018).

Sedatives such as benzodiazepines and opioids are commonly used to alleviate preoperative anxiety. However, these pharmacologic agents have side effects such as drowsiness, respiratory distress, nausea, vomiting, and interactions with anesthetics that require close monitoring (Bradt et al. 2013; Graff et al. 2019; Ebneshahidi and Mohseni 2008). More importantly, doubts about the preprocedural efficacy of commonly used drugs have increased the trend toward nonpharmacologic applications (Graff et al. 2019; Conway et al. 2022; Hole et al. 2015).

Music is one of the most widely used, harmless, inexpensive, and non-pharmacological interventions (Bae et al. 2023). Music applications are considered safe and effective methods for reducing anxiety by inducing pleasure in the individual through their effects on the limbic system (Firmeza et al. 2017; Leardi et al. 2022). Music provides comfort and familiarity, helping patients overcome emotional and physical alienation, and shifting their focus away from current problems, thereby reducing anxiety. From 1914 to the present, clinicians have found music to be relaxing for patients (Firmeza et al. 2017). The choice of genre (McClurkin and Smith 2016) or specific pieces is left to the individual (Kavak Akelma et al. 2022). Perioperative anxiety has been evaluated in numerous studies.

In our previous study, where patients listened to their favorite music during the preoperative period without genre restrictions, we found that postoperative anxiety decreased compared to the control group (Kavak Akelma et al. 2022). This finding led us to hypothesize whether listening to one's favorite preoperative music makes a difference compared to classical music, which is widely accepted in the literature to reduce anxiety, and whether it contributes to the quality of postoperative recovery. In the current study, the primary outcome was to compare the effect of patient-preferred music preference on anxiety with that of classical music in patients undergoing inguinal hernia repair surgery. Secondary outcomes included quality of recovery, pain, patient satisfaction, and hemodynamic parameters.

## Materials and methods

### Study population

After approval by the Ethics Committee of Dışkapı Yıldırım Beyazıt Training and Research Hospital (Ethics Committee 20.01.2020 No. 80/07), this prospective, randomized, controlled, single-blind study was planned and registered with ClinicalTrials.gov (registration number NCT04277559). Written and verbal informed consent was obtained from all patients in accordance with the Declaration of Helsinki. The Consolidated Standards of Reporting Trials (CONSORT) flow chart was used for patient enrollment and allocation. The study was conducted between November 2021 and July 2022 in the operating room of the general surgery clinic of Dışkapı Yıldırım Beyazıt Training and Research Hospital.

Patients between 18 and 70 years of age, American Society of Anesthesia (ASA) physical status I–II, who could speak and understand Turkish language clearly, who could read and write, and who were scheduled to undergo elective inguinal hernia surgery under general anesthesia were planned to be included in the study. Patients with severe hearing impairment, visual impairment, history of narcotic use, alcohol dependence, dementia, regular use of antidepressants, and no specific music preferences were excluded.

**Fig. 1:**
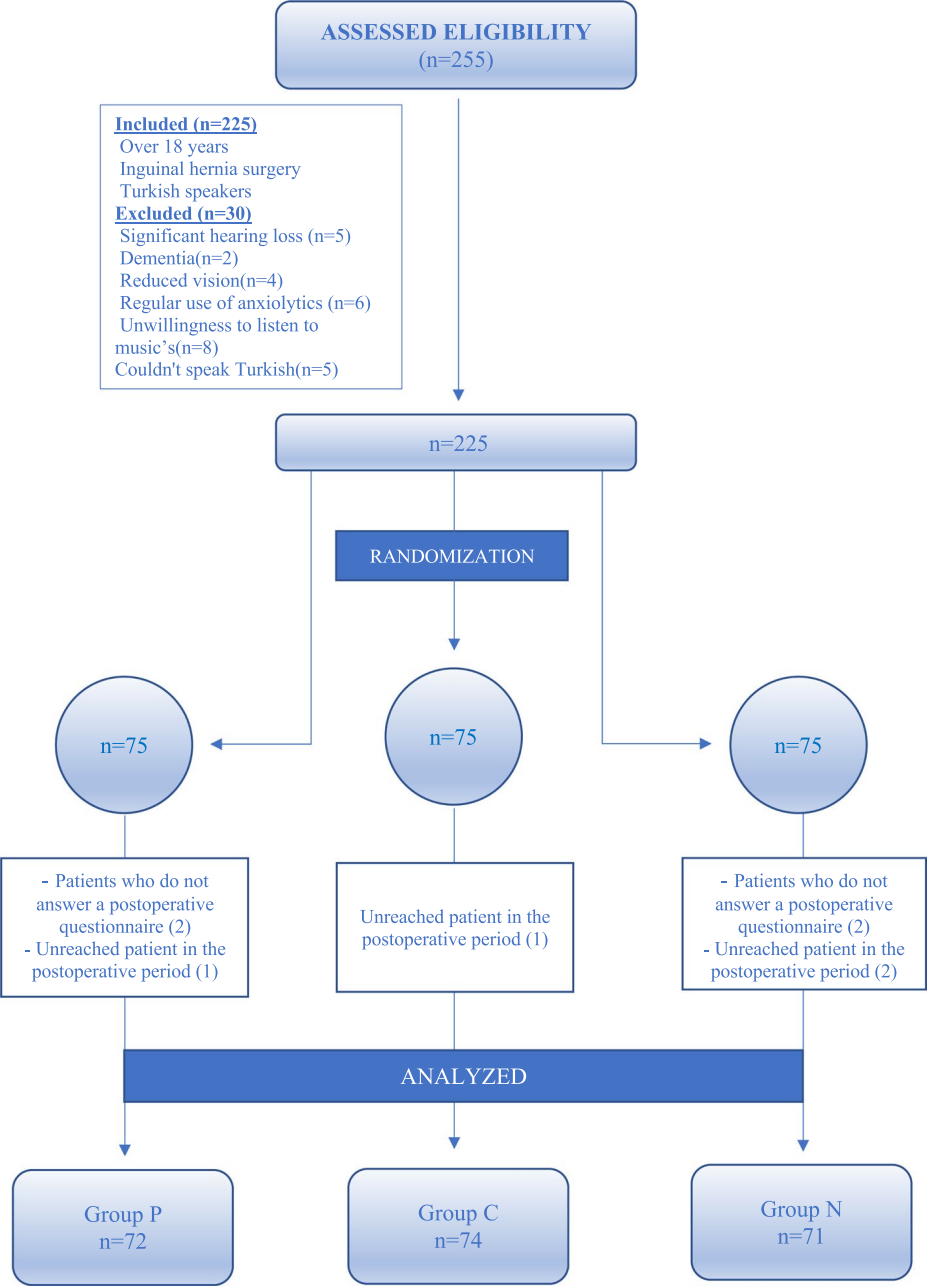
Flow chart

A total of 255 patients were included in the study. Of these, 30 patients were excluded because they did not meet the inclusion criteria. The 225 patients enrolled in the study were randomized into 3 groups by a computerized randomization method patient-preferred music group (group P), classical music group (group C), or no music group (group N) (Fig. 1).

### Preoperative management and intervention

Demographic data such as age, sex, height, weight, previous elective and/or emergency surgery, educational status, and marital status of the patients were recorded during the preoperative evaluation period. Patients were given the STAI-1 and STAI-2 questionnaires to self-report their current state of anxiety and the QoR-40 (40–200 points) questionnaire in their beds 1 h before surgery.

Patients were brought to the waiting room where preoperative measurements were taken. Patients were asked for their favorite music title and singer, without limiting the type of music, and were enrolled in group P. Patients in the preferred music group listened to their favorite music for 15 min. Patients in the classical music group also listened to Vivaldi’s Four Seasons for 15 min, which has been shown to be effective in similar anxiety studies (Firmeza et al. 2017; Mammarella et al. 2007). These two groups of patients used headphones that completely covered their ears to further isolate them from environmental stimuli. The volume of the music was adjusted to remain between 50 and 60 dB (Kavak Akelma et al. 2022). During the same period, patients in the control group did not listen to music or use headphones.

In groups P and C, heart rate and systolic-diastolic blood pressure were measured before and after listening to music. In the control group, heart rate, systolic-diastolic, and mean blood pressure were measured and recorded upon entering the preoperative waiting room and 15 min later. None of the patients were given premedication prior to the procedure. After this procedure, the patients were taken to the operating room.

### Intraoperative management

HR, SBP, DBP, MAP, peripheral oxygen saturation, and bispectral index (BIS Quatro sensor and BIS VISTA monitor) were monitored and recorded noninvasively in the operating room. After induction of anesthesia with fentanyl citrate (1–2 μg/kg) and propofol (2–4 mg/kg), a laryngeal mask airway was inserted. Anesthesia was maintained with sevoflurane (1–1.5% end-tidal concentration) in a 50% air/50% oxygen mixture. The sevoflurane concentration was titrated to a BIS value of 40 to 60. Hypotension was defined as a decrease in MAP of at least 20% from baseline and was treated with intravenous ephedrine 5 mg. Bradycardia was defined as a heart rate of less than 45 beats per minute and was treated with intravenous atropine 0.5 mg. Surgery was performed by an experienced surgeon. All patients underwent open anterior mesh repair. After induction of anesthesia, an erector spinae plane block with 20 mL of 0.25% bupivacaine was performed unilaterally at the T7 level on the hernia side. For analgesia, 1000 mg intravenous acetaminophen and 100 mg intravenous tramadol were administered to all patients approximately 15 min before the end of surgery as part of multimodal analgesia management. In the postoperative period, 50 mg dexketoprofen-trometamol was administered intravenously every 8 h. 50 mg intravenous tramadol was administered as rescue analgesia when the NRS was 4 or higher.

### Outcomes

**Fig. 2:**
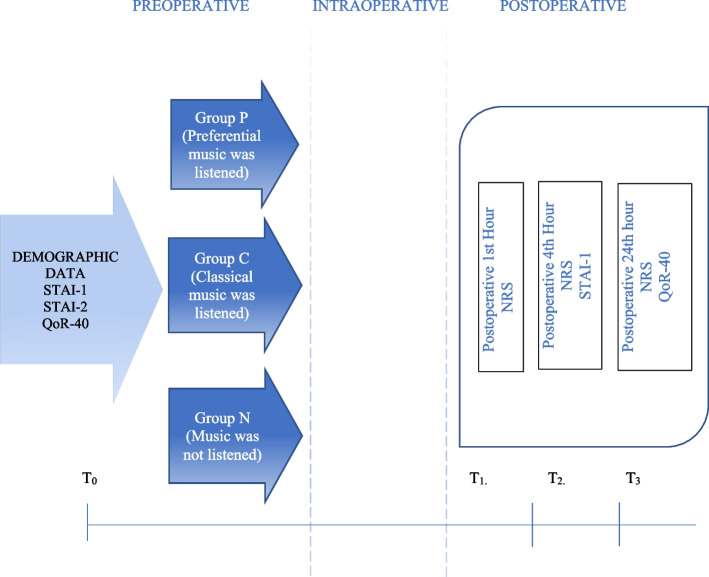
Timeline

The State-Trait Anxiety Inventory (STAI-1 and STAI-2) was determined as the primary outcome of our study. STAI-1 and STAI-2 questionnaires were administered to all patients in their rooms 1 h before surgery, and the STAI-1 questionnaire was repeated 4–6 h after surgery (Fig. 2). The State-Trait Anxiety Inventory (STAI), developed by Spielberger et al. in 1970 (Spielberger et al. 1983) and adapted to Turkish by Öner and Le Compte in 1983 (1983), is a 4-point Likert self-report scale consisting of a total of 40 items, 20 of which are assigned to the state anxiety subscale (STAI-1) and 20 to the trait anxiety subscale (STAI-2). Scores ranged from 20 to 80, with higher scores indicating higher levels of anxiety.

One of the secondary outcomes of our study was the 24-h Quality of Recovery-40 Questionnaire (QoR-40) (Fig. 2). The QoR-40 was defined to assess the quality of recovery of patients in the early postoperative period from the patient’s perspective. The questionnaire includes five parameters: physical comfort (*n* = 12), patient support (*n* = 7), physical independence (*n* = 5), pain (*n* = 7), and emotional state (*n* = 9). The questionnaire consists of a total of 40 questions, and each question is scored on a 5-point Likert scale. These scores are summed to give a total score. The worst collection scores 40 points and the best collection scores 200 points (Myles et al. 2023; Myles et al. 2022). The QoR-40 has been used and validated in people from many different countries with cultural and physical differences (Karaman et al. 2023). Hemodynamic variables recorded before and after the musical performance were evaluated in the study groups and before entering and leaving the waiting room in the control group.

The NRS (numerical rating scale) was used to assess patient pain (0 = no pain; 10= worst pain imaginable). Postoperative NRS scores were recorded at 1, 4, 12, and 24 h (Fig. 2). The Likert scale was used to assess patient satisfaction. The Likert scale consisted of a questionnaire asking for numerical values between 1 and 7. All of these scales were administered by a blinded investigator.

### Statistical analysis

We performed *a priori* sample size calculation, based on pilot data obtained from postoperative STAI-1 scores, using the G*Power version 3.1.9.2 (© Franz Faul, Edgar Erdfelder, Albert-Georg Lang, and Axel Buchner, 2006, 2009) (Faul et al. 2007; Erdfelder and FAul F, Buchner A, Lang AG. 2023). In the pilot study we conducted with 10 patients from each of the 3 groups, the postoperative STAI score was recorded. A sample size of 65 patients in each group was calculated with a power of 95% and an α-level of 0.05 error, 0.285 effect size. To account for a potential 15% attrition rate, a sample of 75 participants per group was required for this study.

Data analysis was conducted using IBM SPSS 25.0 (Armonk, NY: IBM Corp.) and MedCalc 15.8 (MedCalc Software bvba, Ostend, Belgium) statistical package programs. The normality of the distribution of continuous variables was evaluated using the one-sample Shapiro-Wilk test. Patient demographics and characteristics were expressed as number and percentage, median (interquartile range, IQR), and mean (standard deviation, SD), and were analyzed using the chi-square test for categorical variables and the independent *t*-test for normally distributed continuous variables. Mann–Whitney *U* test and Kruskal-Wallis were applied for comparisons of non-parametric and non-normally distributed data. Nominal data were analyzed by Pearson chi-square or Fisher’s exact test where appropriate. The corrected Bonferroni test was used for multiple comparisons. *p* values < 0.05 were considered statistically significant in each test.

## Results

A total of 255 patients were screened for eligibility and 225 patients were enrolled. Three patients in group P, one in group C, and four in group N were excluded because they refused to participate in the study. Consequently, 217 patients (group P; *n* = 72, group C; *n* = 74, group N; *n* = 71) completed the study (Fig. 1).

The groups were similar with respect to age, sex, marital status, body mass index (BMI) and ASA classification, educational status, history of previous surgery, presence of comorbidity, and mean waiting time (*p* > 0.05 for all). Preoperative STAI-2 scores were also similar between groups (*p* = 0.274) (Table 1).

**Table 1:** Demographic data

Variable	Group P (*n* = 72)	Grup C (*n* = 74)	Group N (*n* = 71)	*p* value
Age (year)	37.48 ± 14.01	38.13 ± 11.15	37.43 ± 15.58	0.64
Body mass index (kg/m^2^)	28.35 ± 4.91	28.47 ± 5.07	28.53 ± 4.97	0.97
Gender (female/male)	8/64	12/62	10/61	0.66
Marital status (married/single)	38/34	40/34	32/39	0.50
Educational achievement level (*n*) (%)				
Primary	22 (30.6)	20 (27.0)	17 (23.9)	0.78
Secondary	29 (40.3)	25 (33.8)	24 (33.8)	
High school	17 (23.6)	22 (29.7)	24 (33.8)	
University	4 (5.6)	7 (9.5)	6 (8.5)	
Previous surgery (*n*) (%)				
No	21 (29.2)	23 (31.1)	29 (40.8)	0.41
Elective	17 (23.6)	19 (25.7)	19 (26.8)	
Emergency	18 (25.0)	14 (18.9)	15 (21.1)	
Elective+emergancy	16 (22.2)	18 (24.3)	8 (11.3)	
ASA (1/2/3) (n)	40/28/4	42/30/2	44/25/2	0.81
Presence of comorbidity (yes/no) (*n*)	32/40	30/44	29/42	
Waiting time	21.02 ± 1.41	20.89 ± 1.41	21.09 ± 1.43	0.67
STAI-2 score	40.72 ± 7.15	40.24 ± 6.51	41.42 ± 4.41	0.27

*STAI-2* State-Trait Anxiety Inventory-2

The data are presented as mean ± SD, number (%) or (*n*)

Preoperative STAI-1 scores were similar between groups (*p* = 0.872). Postoperative STAI-1 scores were significantly lower in group P than in group N (*p* = 0.025). There was no statistical difference between postoperative STAI-1 scores when group C was compared with group P and group N (*p* = 0.134 vs. *p* = 1.000, respectively) (Table 2). The change in anxiety score in the postoperative period was statistically significantly higher in group P compared to group N (group P: 6 (5–7); group C: 5 (3–7); group N: 4 (3–5)) (*p* = 0.001) (Table 2). Pre- and post-operative QoR-40 scores showed different results, with group P having a significantly higher post-operative QoR-40 score than group N (*p* = 0.003) (Table 2). There was no statistical difference between postoperative QoR-40 scores when group C was compared with group P and group N (*p* = 0.065 vs. *p* = 0.912, respectively) (Table 2).

**Table 2:** Pain and anxiety scores at baseline and after surgery

Variable	Group P (*n* = 72)	Grup C (*n* = 74)	Group N (*n* = 71)	*p* value
QoR-40 Score				
Before surgery	186.5 (183,190)	186 (182,188.25)	186 (181,190)	0.421
After surgery	185 (177,190) *	182 (176.5,190)	181 (175,184)	0.003
STAI-1 score				
Before Surgery	43.3 (39,50)	45 (41,48)	44 (40,48)	0.872
After Surgery	38 (34.25,43) ^†^	41 (37,43)	41 (37,43)	0.022
Change in anxiety score	6 (5,7) ^§^	5 (3,7)	4 (3,5)	0.001
NRS score				
1 h	2 (2,3)	3 (2,3)	3 (2,3)	0.357
4 h	3 (2,3)	3 (2,4)	3 (2,3)	0.061
12 h	2 (2,3)	2 (2,3)	3 (2,3)	0.143
24 h	1 (1,1)	1 (0.75,1)	1 (1,1)	0.057
Satisfaction scores	7 (6,7) ^‡^	7 (6,7)	6 (6,7)	0.003

*QoR-40 Score* Quality of Recovery Score-40, *STAI-1* State-Trait Anxiety Inventory-1, *NRS* Numerical Rating Scale

The data are presented as median (1Q, 3Q)

^*^According to the Bonferroni correction, Group P was different than Group N (*p* = 0.003)

^†^According to the Bonferroni correction, Group P was different than Group N (*p* = 0.025)

^‡^According to the Bonferroni correction, Group P was different than Group N (*p* = 0.002)

^§^ According to the Bonferroni correction, Group P was different than Group N (*p* = 0.001)

**Fig. 3:**
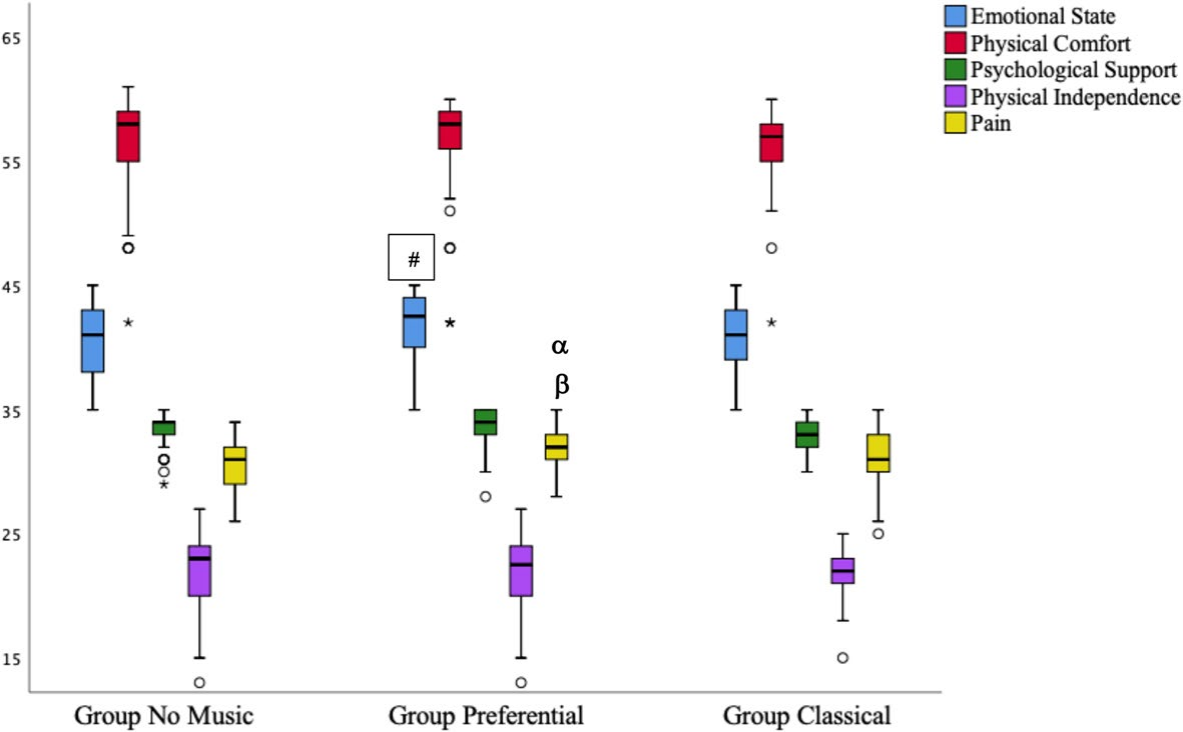
Post-operative QoR 40 Sub-parameters

The physical comfort, physical independence, and patient support scores of the QoR-40 sub-dimensions were not statistically different between all groups, both preoperatively and postoperatively. Emotional state and pain, which are QoR-40 sub-dimensions, did not differ between groups in the preoperative period (*p* = 0.249 and *p* = 0.949, respectively). The postoperative score of emotional state was higher in group P compared to group N (*p* = 0.015), and the postoperative score of pain was lower in group P compared to group C and group N (*p* = 0.043 and *p* = 0.001, respectively) (Fig. 3).

**Fig. 4:**
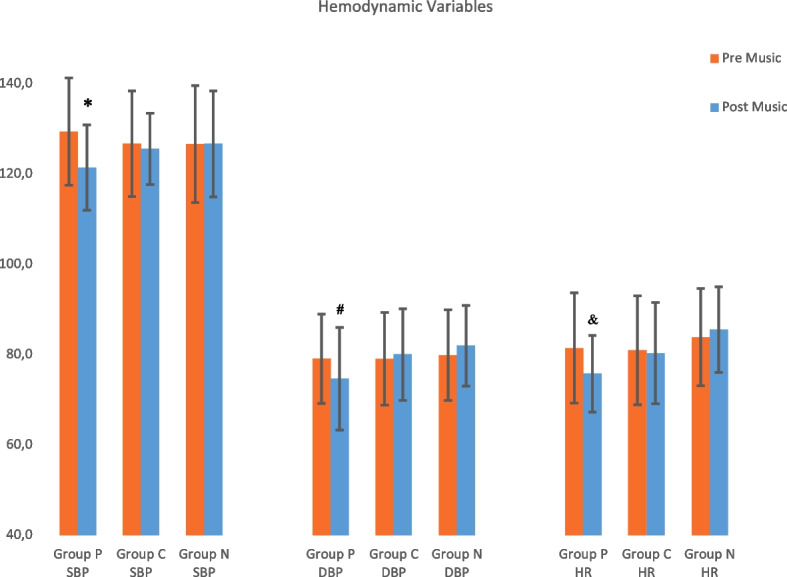
Hemodynamic variables

Baseline levels of SBP, DBP, and HR were similar between groups (*p* = 0.305, *p* = 0.854, *p* = 0.282, respectively). The difference in the amount of change in SBP, DBP, and HR from the pre-music to the post-music assessment was significant between group P (*p* = 0.001, *p* = 0.002 and *p* = 0.001, respectively), and there was no difference in the amount of this change between groups C (*p* 0.330, *p* = 0.498 and *p* = 0.698, respectively) and N (*p* = 0.958, *p* = 0.054 and *p* = 0.733, respectively) (Fig. 4).

NRS scores were not significantly different between groups at any of the assessment time points, i.e. 1, 4, 12, 24 h (*p* > 0.05). Patient satisfaction scores were significantly higher in the P group than in the N group (*p* = 0.003). There was no difference in patient satisfaction scores in group C compared to group P and group N (*p* = 0.099 vs. *p* = 0.564, respectively) (Table 2).

Music preferences in patients in group P varied as follows: (*n* = 19), 26.4% Turkish folk music; (*n* = 19), 26.4% arabesque; (*n* = 12), 16.7% Pop music; (*n* = 13), 18.1% Turkish classical music; (*n* = 19), 4.1% religious content.

## Discussion

This study demonstrated that patient-preferred music is an effective method that contributes to postoperative anxiety and enhances recovery in patients undergoing inguinal hernia repair surgery. To the best of our knowledge, our study is the first to evaluate the contribution of preoperative patient-preferred music on anxiety and postoperative QoR-40 scores. Preoperative listening to classical music, however, did not contribute positively to postoperative recovery. Of note, the utilization of preoperative music did not result in better postoperative pain management.

Many patients develop physical and psychological complaints such as anxiety, depression, aggression, and fatigue when they are scheduled for surgery (Beek et al. 2020). Music, being a universal concept, is appreciated across all ages, cultures, and societies. The type of music listened to varies according to personal preferences as well as social, physical, emotional, and religious parameters. In our previous study, we observed that patients who listened to their favorite music before surgery experienced less postoperative anxiety than those who did not listen to music (Kavak Akelma et al. 2022). In the present study, we found that playing music of one's own choice was more effective in reducing postoperative anxiety than playing music chosen by someone else. Although classical music is the preferred music genre for many societies, we concluded that it would be wrong to include all societies in this (Ruud 2022). It is thought that listening to a patient-preferred piece of music makes us familiar with our past, memories, and social connections.

Although the mechanism is not clearly known, preoperative interventions such as music contribute to the reduction of postoperative anxiety. Researchers found that patients who listened to music before surgery had lower postoperative anxiety, suggesting that music induces a relaxation response and a sense of well-being that reduces sympathetic nervous system activity both preoperatively and postoperatively [23962572]. High serum levels of stress hormones such as cortisol are associated with anxiety and psychological stress, and listening to music before surgery has been shown to reduce serum cortisol levels regardless of gender [24348454]. The presence of preoperative anxiety can affect postoperative anxiety, patient satisfaction, pain, and recovery process.

Postoperative recovery is a complex process involving various parameters such as physiological changes due to anesthesia and surgery, side effects, pain, changes in psychological status, and patient satisfaction (Stamenkovic et al. 2018). There are no studies evaluating the quality of recovery using the QoR-40 questionnaire of preoperative music applications. There are studies that evaluate recovery using different methods. Nilson et al, in a study in which they played intraoperative soothing and relaxing sounds for hysterectomy, as well as sounds with a male voice containing relaxing and encouraging suggestions, examined pain scores, nausea, vomiting, bowel function, fatigue, well-being, and hospital stay outcome variables. They found that the use of music had a positive effect on postoperative pain, fatigue, and postoperative recovery (Nilsson et al. 2022).

In the model created by Kopp et al. using regression analysis to assess postoperative recovery, it was suggested that preoperative state anxiety did not directly affect recovery parameters (Kopp et al. 2003). Postoperative recovery was reported to be improved in the patient group with high social support in daily life, and applications reminding of past good memories and family ties improved postoperative recovery (Stamenkovic et al. 2018; Kopp et al. 2003). In our study, the QoR-40 score was similar in the group that listened to classical music and the group that did not listen to music, while it was significantly higher in the group that listened to patient-preferred music compared with the other two groups. When analyzing the QoR-40 sub-parameters across groups, we observed a higher emotional state assessment score in group P compared to the other two groups. We assessed that the difference in emotional state assessment could be due to increased anxiety levels in the patients. This higher emotional state sub-parameter in Group P is believed to be the primary reason for the higher overall QoR-40 score.

Preoperative anxiety leads to high postoperative anxiety, pain, and prolonged hospital stay (Hole et al. 2015; Caumo et al. 2022). In addition, preoperative anxiety has been shown to adversely affect anesthesia induction and recovery (Gras et al. 2022). In a study evaluating the anxiety and recovery quality of lorazepam given for premedication, it was found that decreased anxiety levels had no direct effect on recovery (Mijderwijk et al. 2013). In a study evaluating the effect of midazolam as an anxiolytic on postoperative recovery, midazolam did not contribute to postoperative anxiety compared to the placebo group (Beek et al. 2020). The study found that the postoperative QoR40 score was similar in the placebo and midazolam groups and suggested that the effect of anxiolytic applications with few sedative side effects on the quality of postoperative recovery should be compared with ERAS applications. However, to our knowledge, no study has shown that effective reduction of preoperative anxiety improves the QoR40 score. In our study, we found that preoperative music application effectively reduced anxiety and consequently increased the QoR 40 score, thereby improving recovery.

Modern theories of pain suggest that the experience of pain is influenced by both physical and psychological factors. It is claimed that cognitive activities, such as listening to music, can influence the perceived intensity and unpleasantness of pain, leading to a reduction in the patient's perception of pain (Hole et al. 2015). Abdul Hamid et al (Abdul Hamid et al. 2022), in their study evaluating the effects of intraoperative music on pain, found that pain scores decreased as long as they listened to the music in the postoperative period, and they recommended music therapy as an alternative to pharmacological applications to reduce anxiety in operating rooms. In a study using music application during epidural catheter insertion for labor, they found that music did not contribute to pain and patient satisfaction (Drzymalski et al. 2017). In our study, we found that hemodynamic data and anxiety improved in the group listening to their preferred music, but there was no difference from the control group in the group listening to classical music. Although music had a positive effect on anxiety and increased satisfaction, it did not contribute to a reduction in pain scores. The classical music group had similar pain scores to the control group and the preferred music group. This shows that effective multimodal analgesia can avoid being influenced by changes in anxiety. Although the patient-preferred music intervention did not affect the NRS score in our study, the pain section of the QoR-40 sub-analysis was additionally examined. In group P, the pain sub-analysis, which is one of the parameters constituting the total QoR-40 score, was observed to be lower. Although we do not have conclusive evidence on this matter, the current situation suggests, as emphasized by Kain et al. (Kain et al. 2001), that reducing preoperative anxiety may prevent a clear postoperative pain response, especially if postoperative pain is not severe. We believe that the difference in pain assessment in the QoR-40 may be attributed to its more detailed nature compared to the NRS and the fact that it was evaluated at the 24th hour.

The study had several limitations. Music preferences may vary depending on societies and cultures. We planned this study to compare patient-preferred music to classical music in Turkish society patients. One of the limitations of our research is that it was applied to a limited ethnic group. Another limitation of this study is that the patient profile included a limited group of patients who underwent a single type of minor surgery. To generalize the results, different types of surgeries should be added in future studies. Furthermore, there is also a need for studies of anxiety in major types of surgery.

## Conclusion

Listening to preferred music before inguinal hernia repair surgery has been shown to reduce anxiety and improve the quality of recovery compared to classical music. As can be seen from our findings, a decrease in anxiety score (38 (34.25,43), 41 (37.43), respectively) and an increase in postoperative recovery score (185 (177,190), 182 (176.5,190), respectively) were detected in the preferential music group compared to the classical music group. This can also contribute to clinical indicators such as hemodynamic data and patient satisfaction. Our findings from the Turkish community indicate that patient-preferred music is more effective than classical music. It is believed that this may be due to differences in perspectives on music due to social variations.

## Data Availability

No datasets were generated or analysed during the current study.
